# Chemotherapy-Related Differences in Cognitive Functioning and Their Biological Predictors in Patients with Multiple Myeloma

**DOI:** 10.3390/brainsci11091166

**Published:** 2021-09-01

**Authors:** Magdalena Bury-Kamińska, Aneta Szudy-Szczyrek, Aleksandra Nowaczyńska, Olga Jankowska-Łęcka, Marek Hus, Klaudia Kot

**Affiliations:** 1 Department of Clinical Psychology and Neuropsychology, Institute of Psychology, Maria Curie-Skłodowska University in Lublin, 45 Głęboka, 20-612 Lublin, Poland; 2 Department of Hemato-Oncology and Bone Marrow Transplantation, Medical University of Lublin, 2 Karmelicka, 20-400 Lublin, Poland; aneta.szudy-szczyrek@umlub.pl (A.S.-S.); aleksandra.nowaczynska@umlub.pl (A.N.); olga.jankowska-lecka@umlub.pl (O.J.-Ł.); marekhus@umlub.pl (M.H.); klaudia.kot@umlub.pl (K.K.)

**Keywords:** cancer-related cognitive dysfunctions, cytokines, hematooncology

## Abstract

The paper presents a study on the changes in cognitive functioning in patients undergoing chemotherapy with diagnosed multiple myeloma (MM). The aim of the study was to answer the following two main research questions: Does the treatment stage differentiate the functioning of cognitive processes in patients with diagnosed MM and to what extent? Is it possible to treat biological factors (TNF-α, IL-6, IL-10, and BDNF) as predictors of patients’ cognitive functioning? The patients were examined twice, before the treatment and after 4–6 cycles of chemotherapy. Selected neuropsychological research methods as well as experimental and clinical trials were employed to diagnose the patients’ general cognitive state, attention, memory, and executive functions. The level of biological factors was assessed with the ELISA test. The results show that the patients’ cognitive functioning was worse before the treatment than during the cytostatic therapy. It was also possible to predict the cognitive state of patients suffering from multiple myeloma based on a selected biological parameter (neurotrophin BDNF).

## 1. Introduction

The research reports to date corroborate the existence of changes in the cognitive functioning of patients suffering from cancer in response to oncological treatment [1,2,3,4]. This phenomenon is heterogeneous in character [5]. Thus, in order to increase the coherence of the data connected with the occurrence of cognitive changes in patients undergoing anti-cancer treatment, the common aim is to study credible and reliable causes of cancer-related cognitive dysfunctions (CRCD).

Cognitive dysfunctions do not occur in all patients with the same intensity and frequency. However, the dysfunctions of memory, attention, and executive functions are most burdensome to them [6,7].

According to recent research, one of the most important factors influencing the occurrence of cognitive changes due to oncological treatment is the dysregulation of cytokines [8], that is, IL-6 (interleukin 6) [9], IL-10 (interleukin 10) [10], and TNF-α (tumor necrosis factor) [9,11]. Brain-derived neurotrophic factor (BDNF) [12] may also have a modifying influence on cognitive functions.

It was demonstrated that the neoplastic process increases the level of cytokines, that is TNF-α, IL-6, and IL-10, which have an impact on cognitive changes [13,14].

Cognitive impairments also occur in patients with plasma cell myeloma, also known as multiple myeloma (MM) [15,16], defined as a clonal proliferation of abnormal plasma cells. Studies show that the diversified levels of cytokines may be one of the etiological factors of MM [17,18].

Possessing the knowledge concerning the likely causes of CRCD in other types of cancer, and taking into consideration that the etiological factors of cognitive changes in oncological treatment are IL-6, IL-10, BDNF, and TNF-α, it is logical to propose a hypothesis that a relationship exists between the advancement of a neoplastic process, the level of enumerated biological factors, and the level of cognitive functioning in patients with MM.

## 2. Materials and Methods

In total, 21 patients diagnosed with MM underwent the examination. The exclusion criteria were an oncological, neurological, and/or psychiatric disease in the past. These were usually people in their 7th decade of life (M = 65, SD = 8.5). Women constituted a slight majority in comparison to men (F = 57%, M = 43%). The average educational period in the life of the studied patients was 15 years (SD = 5). The studied patients underwent chemotherapeutic treatment based on two or three drugs: VCD–bortezomib, cyclophosphamide, and dexamethasone (33%), VTD–bortezomib, thalidomide, and dexamethasone (62%), or VD–bortezomib and dexamethasone (5%).

The methods used in the research are in line with the objectives of evidence-based psychological practice (EBPP). These included the following:

1. Montreal Cognitive Assessment (MoCA)—a screening tool comprising tests assessing various aspects of cognitive functions [19].
2. Battery of Tests for Assessing Cognitive Functions PU1—a comprehensive tool for assessing memory, attention, and executive functions. In this research, it was used in an experimental way, and the following subtests were selected: Deferred Naming Test, assessing working memory; Attention Divisibility Test, measuring the ability to divide attention between verbal and auditory material; Park Map Test, assessing planning abilities; Verbal Fluency I (letter) and II (semantic) [20].
3. The experimental and clinical trials based on Choynowski’s Memory Scale are a research method based on the Wechsler Memory Scale (WMS). The following subtests were used: Short-term Auditory Memory; Numbers Directly, measuring short-term memory; Numbers Backwards, assessing the functioning of working memory; Long-term Memory [21].
4. Stroop Color–Word Interference Test (SCWT), used in an experimental way to assess executive functions. The indicators of completion are the execution time and the number of errors. The test involves three tasks: (1) Color Naming; (2) Reading Color Names; (3) Interference Task: a measure of execution control [22].
5. Depression Measurement Questionnaire, measuring the level of depression [23].

The authors’ own research is also based on a laboratory diagnosis of the following biological parameters in the patients’ blood serum: TNF-α, IL-6, IL-10, and BDNF. Their indicator is the concentration of these compounds in the patients’ blood serum measured in picograms per milliliter (pg/mL). The level of cytokines was marked in blood serum using an immunoenzymatic method—a Quantikine High-Sensitivity enzyme-linked immunosorbent assay (ELISA) by R&D Systems. The blood serum was sampled from clotted peripheral blood.

All patients underwent blood tests in the same conditions—after a period of fasting, early in the morning. The test material was approximately 3 mL of peripheral blood samples collected to serum gel tubes. The samples were centrifuged at 3000 rpm for 10 min. The clot was removed by centrifugation to obtain more than 1 milliliter of blood serum. The serum was pipetted into tubes and stored at −80 °C until further analysis. It was the so-called absence of duplicates.

The aim of this research study is to explain the causes of a great diversity of negative effects of cancer and oncological treatment in the form of cognitive changes. An attempt was made to verify the hypothesis concerning the crucial meaning of biological factors, such as cytokines TNF-α, IL-6, and IL-10, as well as neurotrophin BDNF, whose level may modify cognitive changes in patients.

The research procedure took place at the Hematooncology and Bone Marrow Transplantation Clinic. Apart from neuropsychological tests, the levels of cytokines IL-6, IL-10, and TNF-α, as well as neurotrophin BDNF, were also analyzed. The patients underwent the research procedure twice, each time in a single day. Considering the inconsistencies connected with the period of symptom persistence in patients with CRCD [24,25], the authors decided to take the measurements of cognitive, biological, and psychosocial variables twice—before chemotherapeutic treatment (T0) and after 4–6 cycles from the start of anti-cancer therapy (T1).

The answers provided by the patients were subject to statistical analysis with the use of SPSS Statistics Version 25 and the arithmetic mean and standard deviation were used. In order to assess the differences in the results obtained from the comparison of two groups, a t-test for the dependent samples was used. The t-test took into consideration the non-homogeneous variances in the case of some variables. The subsequent step of the analysis was to determine the predictors of selected cognitive functions in both measurements. For this purpose, linear regression analysis was conducted. Due to the small sample size (*n* = 21), the regression model was made separately for each predictor.

The research protocol was approved by a bioethical committee appropriate for the institution where the research took place (no. KE-0254/253/2016). All patients who were part of this research gave their written informed consent for their participation in the study and for the use of their test results for the purpose of this research. The authors archived patients’ written consent forms.

## 3. Results

The following research question was asked: When and how does cognitive dysfunction occur in patients with MM? The results obtained from before the chemotherapeutic treatment and after 4–6 cycles of chemotherapy were compared.

As a result of analyses, a difference at the level of statistical significance concerning the general cognitive functioning measured by MoCA was observed (t(20) = −1.989; *p* = 0.061). In the first measurement, the result was higher (M = 21; SD = 3.25) in comparison to the second measurement (M = 23; SD = 2.87). This means that the studied patients had better cognitive functioning during the treatment than before it. It should also be highlighted that test results at the level of statistical tendency are marginally significant and strong conclusions cannot be drawn on their basis. What is more, the tests were carried out on a Polish sample that does not have the standardized MoCA norms. This is why the authors decided to apply data analyses involving the raw results. However, knowing the cut-off point for the normative results of MoCA (26 points), it turns out that during the first examination, only four patients (19% of all participants) obtained a result that was the same or higher. In turn, during the second examination, a normative result was obtained by three patients (14% of all participants). It may indicate that a significant majority of the sample obtained results that are worse than normative within the scope of general cognitive functioning before chemotherapy and during the cytostatic treatment.

Selected subtests of the Battery PU1 were other tools that were implemented. A difference at the level of statistical tendency was noted only within the scope of the Attention Divisibility Test, which measures the ability to divide attention between verbal and auditory material (t(20) = −2.023, *p* = 0.057). The ability to select information and stimuli from more than one source and focus attention on at least two aspects of the environment at the same time was better after 4–6 treatment cycles (M = 5.33; SD = 2.24) in comparison to the beginning of treatment (M = 4.52; SD = 1.5). Test results at the level of statistical tendency that are marginally significant cannot be treated as a basis to draw strong conclusions.

Regarding the experimental and clinical trials that measured different memory aspects, results that considerably differentiated the studied patients during the two measurements were obtained in terms of short-term auditory memory (t(20) = −2.278, *p* ≤ 0.05) and long-term auditory memory (t(20) = −2.278, *p* ≤ 0.05). The ability to store auditory information in short-term memory was significantly better during the chemotherapeutic treatment (M = 6.57; SD = 2.54) in comparison to the first examination (M = 5.14; SD = 2.56). A Cohen’s d value of 0.5 indicates a medium effect size. A similar tendency can be observed in the case of the analysis of long-term auditory memory (t(20) = −1.948, *p* = 0.066). The patients also achieved better results during the second measurement (M = 5.67; SD = 2.85) in comparison to the pre-treatment period (M = 4.33; SD = 2.6). The authors cannot treat test results at the level of statistical tendency that are marginally significant as a foundation for strong conclusions.

Regarding the indicator of lexical fluency and lexical access speed—one of the measures of the SCWT—differences at the level of statistical significance were observed (t(20) = 2.118, *p* ≤ 0.05). This means that the studied patients scored better during the repeated measurement, as they made fewer errors (M = 0.29; SD = 0.56), than at the beginning of the anti-cancer therapy (M = 0.76; SD = 1.14). A Cohen’s d value of 0.46 indicates a medium effect size between the two examinations.

In order to control for the influence of demographic variables (age, education) during the first examination (before chemotherapy), the authors determined if there is a significant correlation between particular parameters of cognitive functions. The following indicators significantly correlate with the age variable: PU1, Deferred Naming Test—number of named images (r = −0.629; *p* ≤ 0.01); PU1, Deferred Naming Test—number of starts (r = 0.607; *p* ≤ 0.01); PU1, Park Map Test—execution time (r = 0.567; *p* ≤ 0.01); PU1, Verbal Fluency II—number of words (r = −0.505; *p* ≤ 0.05); PU1, Verbal Fluency II—number of clusters (r = −0.632; *p* ≤ 0.01); PU1, Verbal Fluency II—number of switches (r = −0.568; *p* ≤ 0.01); SCWT, Color Naming—execution time (r = 0.496; *p* ≤ 0.05); SCWT, Interference Task—execution time (r = 0.576; *p* ≤ 0.01). In addition, the following indicators significantly correlate with years of education: PU1, Attention Divisibility Test—mathematical operations (r = 0.447; *p* ≤ 0.05); PU1, Park Map Test—execution time (r = −0.44; *p* ≤ 0.05); SCWT, Interference Task—execution time (r = −0.436; *p* ≤ 0.05). This may be proof that the influence of biological parameters (such as cytokines and neurotrophin BDNF) on patients’ cognitive state is non-exclusive. The cognitive state may be additionally modified by demographical variables.

Similar calculations were carried out for the second examination (after 4–6 cycles of chemotherapy). They showed that the following indicators significantly correlate with the age variable: PU1, Park Map Test—execution time (r = 0.663; *p* ≤ 0.01); PU1, Verbal Fluency II—number of words (r = −0.61; *p* ≤ 0.01); PU1, Verbal Fluency II—number of clusters (r = −0.51; *p* ≤ 0.05); PU1, Verbal Fluency II—number of switches (r = −0.5; *p* ≤ 0.05); Choynowski’s Memory Scale—short-term auditory memory (r = −0.482; *p* ≤ 0.05); SCWT, Interference Task—execution time (r = 0.451; *p* ≤ 0.05). In addition, the following variables significantly correlate with the years of education: PU1, Verbal Fluency II—number of words (r = 0.544; *p* ≤ 0.05); PU1, Verbal Fluency II—number of switches (r = 0.511; *p* ≤ 0.05). The above-mentioned correlations may be an indicator of more than one influence of biological parameters on the level of patients’ cognitive functioning. It may be additionally modified by patients’ age and education. It is worth highlighting that the level of depression was a controlled variable in the study. However, its more in-depth analysis exceeds the scope of this article.

The second research question regarded the possibility of predicting the cognitive functioning of patients with diagnosed MM on the basis of biological factors (cytokines TNF-α, IL-6, IL-10, and neurotrophin BDNF).

Box plot graphs were used in order to analyze the results. The graphs for the following variables allowed for isolating a maximum of three untypical and extreme values (a max. 14% of all results): BDNF—measurement 1; TNF-α—measurement 2. The outliers were excluded from further analyses.

The correlations between all cognitive functions and biological parameters were tested during the first examination, T0. The analysis involved only those cognitive variables that gave a result at the level of statistical significance or tendency.

The obtained correlation coefficients demonstrated a certain relationship between neurotrophin BDNF and selected cognitive variables, that is, semantic fluency in terms of cluster number (r = 0.557; *p* ≤ 0.05) and SCWT—Color Naming time (r = −0.547; *p* ≤ 0.05).

Subsequently, an analysis of linear regression was carried out. Owing to the small sample size, it was done separately for each dependent variable that is part of the cognitive factors. For the calculation, the level of neurotrophin BDNF was considered the predictor each time. These data are included in Table 1.

The indicator from the Battery PU1—semantic fluency, in terms of cluster number, was the dependent variable, whereas the level of neurotrophin BDNF was the predictor. A statistically significant model (F(1,16) = 7.196; *p* ≤ 0.05) with a statistically significant predictor (t = 2.683; *p* ≤ 0.05) was obtained. On the basis of a higher level of BDNF, it is possible to predict a better implementation of semantic strategies before the treatment.

The subsequent indicator that was analyzed comes from the SCWT—Color Naming time. A statistically significant model (F(1,16) = 6826; *p* ≤ 0.05) with a statistically significant predictor (t = −2.613; *p* ≤ 0.05) was obtained. On the basis of the above-mentioned data, it is possible to draw a conclusion that the increase in the BDNF level helps in predicting a shorter color naming response time—one of the indicators of executive functions.

Similar statistical analyses were implemented for the measurement carried out after 4–6 treatment cycles. The correlations between all cognitive functions and biological parameters during the retest were analyzed.

The biological variable, that is, the predictor (BDNF), is correlated with the variable described (selected cognitive function) at the level of statistical tendency. A relationship between the level of biological factor BDNF, short-term visual memory indicator (r = 0.401, *p* = 0.072), and working memory indicator (r = −0.416, *p* = 0.061) was noted.

The subsequent stage was the linear regression analysis. Owing to the small sample size, it was done separately for each dependent cognitive variable.

On the basis of the data in Table 2, it can be concluded that a statistically significant model (F(1,19) = 3.634; *p* = 0.072) and a statistically significant predictor BDNF (t = 1.906; *p* = 0.072) have been obtained. The increase in BDNF influenced the higher number of memorized and recreated elements in the short-term visual memory inventory after 4–6 cycles. Another regression model that is well-matched to the set-up of the analyzed variables (F(1,19) = 3.965; *p* = 0.061) indicates that the increase in the BDNF level results in fewer reattempts. It reflects better functioning of short-term visual memory. It is worth pointing out that test results at the level of statistical tendency are marginally significant. Thus, it is impossible to draw an unambiguous conclusion.

## 4. Discussion

The authors’ own research demonstrates a difference in the functioning of patients suffering from MM over the course of chemotherapy. The studied patients were characterized by better functioning within the scope of general cognitive state, attention divisibility, short-term and long-term auditory memory, lexical fluency, and lexical access speed after 4–6 chemotherapy cycles in comparison to the period before the treatment. Apart from few studies [26] describing the occurrence of cognitive changes in patients with MM, there is no accurate data concerning CRCD in this type of cancer. There are reports concerning the occurrence of cognitive dysfunctions in patients after bone marrow transplantation [16,27,28], but not as a result of chemotherapy alone. For comparison, a diminished learning capacity and functioning of auditory memory, as well as the occurrence of cognitive dysfunctions [16], are reported in patients with MM after bone marrow autotransplantation. This is an opposite phenomenon in comparison to the results obtained in the authors’ own research. This can be explained by the fact that bone marrow transplantation is implemented in case of an advanced disease process when chemotherapy alone does not bring the expected effect [29].

The longitudinal studies carried out by Jacobs and associates, in which the studied group mostly comprised individuals with MM (63%), show that the patients’ cognitive functioning improved after one year from the treatment completion. Thus, it can be presumed that CRCD [30] is of transient nature, which is corroborated by this research. Opposing results were obtained by Roman and associates in 2019 [31]. These research results show that the cognitive functioning of patients with MM was insignificantly worse during the treatment in comparison to the control group. These are preliminary reports requiring further analysis.

Learning how to solve neuropsychological tests could have an impact on the improvement of patients’ test results. The authors attempted to control the said impact by introducing a lag between the first and second examinations (around 4–6 months). Another element that was supposed to corroborate the lack of practice effect was the introduction of a pilot study. During the second examination, the patients highlighted multiple times that they do not remember the content of particular tasks. This may be connected with the specificity of their hospitalization. However, it is impossible to explicitly exclude the likelihood of unconscious learning. From a practical standpoint, when analyzing cognitive changes in patients with MM, it is also important to take into consideration the impact of previous experiences with neuropsychological tests.

The second part of the research constituted the verification of the hypothesis concerning the prediction of cognitive functioning in patients with MM on the basis of selected biological parameters. The study showed that before the commencement of the treatment, the BDNF level can help predict selected aspects of cognitive functioning. During the chemotherapeutic treatment, it was also reported that the increase in the BNDF level results in an improved capacity of short-term inventory. When studying the genetic factors of BDNF in women with breast cancer, it turned out that being a BDNF Met allele carrier was a protective factor against CRCD within the scope of self-observed memory dysfunctions, multitasking, and verbal abilities [32]. Therefore, it can be concluded that the BDNF level has a positive influence on memory processes, which is corroborated by the authors’ own research results.

The authors’ own research did not show a relationship between the predictor TNF-α and other cognitive variables. However, the decrease in cognitive functions as a result of an increased TNF-α level can be found in other reports [33,34], mostly in a group of patients with breast cancer whose case results proved inferior memory functioning.

The presented research has a few limitations that ought to be mentioned. The analyzed group of patients is too small to make generalized assumptions about the obtained results in reference to the population of people suffering from MM who undergo treatment.

The limitations of the research also include the lack of a control group. Consequently, it is impossible to definitely establish the extent to which the authors’ own research results are specific to patients with MM and the extent to which they are a manifestation of general social tendencies among individuals without oncological diseases with a similar demographic character.

The studies are also worth enriching with a comparative analysis of the third measurement of cognitive functions and psychosocial variables—after the treatment—so that it is possible to obtain a complete picture of the dynamics of cognitive changes in patients with MM.

Despite the presented limitations, the innovative character of this research stems from a simultaneous consideration of the following in the analyses: the dynamics of cognitive changes in patients at different treatment stages, the measurement of variables in a homogeneous group of patients, and the prediction of the cognitive state on the basis of biological variables.

## 5. Conclusions

On the basis of the research, it is possible to presume better cognitive functioning of patients during chemotherapy (divisibility of attention, short-term auditory memory, long-term memory, and lexical fluency) in comparison to the diagnosis phase. Concurrently, it can be concluded that neurotrophin BDNF can help predict the level of selected cognitive functions already at the diagnosis stage. During chemotherapeutic treatment, BDNF helps predict the capacity of working memory

CRCD may have a different impact on different groups of cancer patients. Until now, the available literature has focused on describing the cognitive changes in patients diagnosed with cancer, also in the form of a tumor, for example, breast cancer. In reference to a hematological disease, the results of the authors’ research may suggest that the cognitive functioning of patients with MM is less dysfunctional than in the case of patients with a solid tumor. The results also suggest that the extent of the dysfunction may occur more slowly, which indicates that the research should be continued. In addition, the long-term effects of chemotherapy in patients with MM should be analyzed as well, especially MM that is characterized by a progressive and chronic nature. This is why, in the course of the disease, patients are prone to more frequent and longer chemotherapy sessions, increased side effects, and symptom burden. Patients with MM are most often elderly people with chronic disease outbreaks in their bones and nephropathic changes, which additionally have impacts on their physical and mental state and functioning.

The results of the planned analyses enhance the understanding of the occurrence and changeability of symptoms of cognitive dysfunctions in a group of oncological patients. Therefore, they will surely enrich the current state of knowledge concerning the causes of CRCD. The results of these studies will contribute to establishing a link between the level of particular biological factors and the selected cognitive functions in patients with MM. This knowledge will have an impact on the improvement of patients’ quality of life.

## Figures and Tables

**Table 1:** Linear regression—first measurement, T0.

Predictor	Dependent Variable	ANOVA (Sig.)	*R*^2^ Value	Standardized Coefficient *β*
BDNF	PU1—Verbal Fluency II cluster number	0.016 *	0.31	0.557
	SCWT—Color Naming time	0.019 *	0.299	−0.547

* *p* ≤ 0.05 is the level of statistical tendency. Source: own research.

**Table 2:** Linear regression—second measurement, T_1_.

Predictor	Dependent Variable	ANOVA (Sig.)	*R*^2^ Value	Standardized Coefficient *β*
BDNF	PU1, Deferred Naming Test— short-term memory indicator	0.072 ***	0.161	0.401
	PU1, Deferred Naming Test— working memory indicator	0.061 ***	0.173	−0.416

*** *p* ≤ 0.1—the level of statistical tendency. Source: own research.

## Data Availability

The data presented in this study are available upon request from the corresponding author. They are not publicly available due to the fact that the data sheet contains information that exceeds the scope of this study and which may be used for other research papers in the future.
